# Exploration of Performance Kinetics and Mechanism of Action of a Potential Novel Bioflocculant BF-VB2 on Clay and Dye Wastewater Flocculation

**DOI:** 10.3389/fmicb.2019.01288

**Published:** 2019-06-07

**Authors:** Varsha Bisht, Banwari Lal

**Affiliations:** ^1^Department of Biotechnology, TERI School of Advanced Studies, New Delhi, India; ^2^Environmental and Industrial Biotechnology Division, The Energy and Resources Institute, India Habitat Center, New Delhi, India

**Keywords:** bioflocculant, eco-friendly, cation-independence, bridging, pseudo first order, wastewater

## Abstract

This study explores production of an efficient bioflocculant; BF-VB2, by strain *Bacillus* sp. TERI VB2 and proposes its potential application in wastewater treatment. One milligram of BF-VB2 can effectively flocculate 1980.0 mg ± 5.0 mg of kaolin particles leading to 99.0% ± 0.5% enhancement in flocculation activity and 99.6% ± 1.0% reduction in turbidity; in less time. BF-VB2 when applied for treatment of textile dyeing industrial wastewater revealed reduction in dye color (82.78% ± 3.03%), COD (92.54% ± 0.24%), TSS (73.59% ± 0.71%), and chloride ions (81.90% ± 0.716%). The best-fit kinetic model (for both COD removal, and dye decolorization) was pseudo-first order with regression coefficient of 0.98 and 0.95, and rate constant of 4.33 × 10^-2^ and 1.83 × 10^2^, respectively. Bridging due to presence of surface charges have been proposed as flocculation mechanism. From results obtained during test-tube studies, flocculation in larger volumes (0.01–5.0 L) was also performed to intend taking up BF-VB2 for *in situ* industrial wastewater treatment. This eco-friendly polysaccharide bioflocculant had longer shelf-life, stability to pH and temperature, cation-independence, and emerged to be more efficient than other flocculants assessed. This study proposed BF-VB2 as a potential natural flocculant candidate for industrial application.

## Introduction

Due to rapid industrialization, water pollution has become a major concern for both government and researchers worldwide (Okaiyeto et al., 2016b). Industrial discharge of wastewater involves various pollutants, such as heavy metals, dyes, suspended particles, sludge, organic matter, aromatic compounds, etc. Dye containing wastewater, specifically is one of the major environmental issue due to: (i) large number of industries (textile, paper and pulp, food, and leather) generating such wastewater; (ii) the dyes are toxic (many of them are carcinogenic, teratogenic, mutagenic) to human beings, aquatic lives, and microorganisms; and (iii) dyes are sometimes recalcitrant to microbial degradation (Yang et al., 2013; Aljeboree et al., 2017).

Textile industries utilizes and in turn releases a lot of water contaminated with dyes and other suspended contaminants. India, is the world’s third largest textile exporter; with textile industry contributing about 11.2% of the national total merchandise export (Restiani and Khandelwal, 2016^1^). In particular, textile industries during the process of dyeing generates a huge amount of effluent containing toxic substances. The solid content is also in the range of 4–5%^2^. Focusing this, it is imperative to develop technologies which are effective in removal of these contaminants and colloidal particles from water and wastewater (Liu et al., 2018). The methods used to treat textile dyeing wastewater involve: coagulation and flocculation, ion-exchange, electrochemical and photo-catalytic degradation, membrane filtration, adsorption-oxidation, and incineration (Yang et al., 2013).

Since long, flocculation (amongst various processes present) has been an essential part of the treatment of wastewater (Shih et al., 2001). Flocculants are used for the flocculation process and they act in the separation of suspended solids from wastewater through charge neutralization, colloid entrapment, and bridging (Lee et al., 2014). A variety of flocculants are available, which include conventionally used: (i) synthetic flocculants (inorganic and organic polymeric flocculants) and comparatively recent (ii) natural flocculants; either of plant origin *viz*. chitosan, starch, cellulose, guar gum, etc., or of microbial origin produced by bacteria, fungi, algae (Lazarova and Manem, 1995; Lee et al., 2014). Though chemically synthesized flocculants have made a mark for themselves due to their cheaper cost and higher efficiency, their non-biodegradable nature and harmful effects on human health (Sathiyanarayanan et al., 2013) have led to a shift in the focus of research toward naturally occurring bioflocculants (both from microbes and plants).

Bioflocculation is an alternative to the conventional flocculation methods, which involves the process of floc (i.e., small or large aggregates of fine particles or solids) formation through microbially produced extracellular polysaccharides (EPS) or bioflocculants (Liu et al., 2009). The bacteria secrete extractable EPS, which being adhesive and cohesive in nature aids in conglomeration of suspended solids present in water or wastewater (Chen and Stewart, 2002). Broadly, various organic substances build-up the bacterial EPS; which includes carbohydrates, uronic acids, proteins, nucleic acids, lipids, etc. (Sun et al., 2012; Nouha et al., 2018). The EPS possess several functions, one being flocculation activity and therefore are used as potential flocculants as they are harmless, biodegradable, and free of any secondary pollutants (Gao et al., 2009; Liu et al., 2009; Sun et al., 2012). Several bacterial species have been reported for their superior flocculating activity, yield, and various additional functions in wastewater treatment, such as biosorption of heavy metals (Salehizadeh and Shojaosadati, 2003); microbial load removal (Buthelezi et al., 2009); dye color removal (Zhang et al., 2007; Li et al., 2013); COD reduction (Zhang et al., 2007; Gong et al., 2008); antioxidant activity (Raza et al., 2012); sludge dewatering (Liu et al., 2014), etc.

It is need of the hour to search for options which are cheaper, energy efficient, and yet effective to mitigate water pollution. Natural flocculants can therefore prove to be a boon for the same. Several plant-based bioflocculants have emerged to be effective in treating wastewater through removal of pollutants. However, moderate activity and shorter shelf-life restricts their feasibility too (Lee et al., 2014). Although bacterial flocculants possess superior properties but still they are deprived of commercialization (Yin et al., 2014). This is attributed to high production cost and low yield. Therefore, to generate commercial exposure of bioflocculant, strategies which focus on developing bioflocculant with low production cost, high yield, longer shelf-life, no additional co-reagent requirement, diverse application, and stability to tackle harsh industrial conditions are required.

Based on the points highlighted above, we report for the effective production of a bacterial flocculant designated as BF-VB2 and its role in treatment of wastewater. In particular, we have emphasized on assessment of this bioflocculant through: (a) its characterization; (b) investigation of its flocculation performance, properties, kinetics, and mechanism of action; (c) comparison of its efficiency with other flocculants; (d) evaluating its role in both clay and dye flocculation; and (e) progressive large volume treatment of wastewater through flocculation process. Results from the study had revealed potential of BF-VB2 in treating highly turbid synthetic wastewater stimulated with kaolin clay at a wide range of pH (4.0–10.0). Further, bioflocculant was assessed for its ability to compete with commercial flocculant (alum) and natural plant-based (lemon) flocculant. Effective flocculation performance of bioflocculant BF-VB2 thus proved to be beneficial in treating water from different sources. The results indicated prospective of BF-VB2 bioflocculant in wastewater (especially dyeing industry) treatment. In addition, BF-VB2 exhibited stability in F.A. (%) till 6 months of examination at room temperature, which denotes its longer shelf-life. Authors have also evaluated a plant-based flocculant for flocculation. With the results obtained in this study, authors are therefore hopeful toward efficiency of BF-VB2 as a potential cleaner bioflocculant and thus, further development of an upscale real wastewater treatment process is in progress.

## Materials and Methods

### Bioflocculant Producing Bacteria and Culture Conditions

EPS producing bacterial strains were isolated from soil sample procured from India Habitat Centre, New Delhi, India (28.5897°N, 77.2249°E). The soil sample was diluted serially; plated on Luria Agar (LA) plates; and incubated at 37°C for 48 h. The bacterial isolates were selected based on the type of colony (ropy, mucoid, and EPS producing) produced on LA plates and were then subjected to morphological assessment.

The purified bacterial isolates were further screened for bioflocculant production using medium composed of (g/L) glucose, 5.0; yeast extract, 1.0; urea, 1.0; KH_2_PO_4_, 0.1; K_2_HPO_4_, 0.1; NaCl, 0.1; MgSO_4_, 0.2 (Xiong et al., 2010). Around 1% of overnight grown seed culture (in Luria Bertani Broth at 37°C, 150 rpm) was inoculated in the above medium, incubated at 37°C, 150 rpm for 120 h, and samples were collected (every 24 h) for assessment. All the initial experiments were conducted in 100 mL Erlenmeyer flask with 50 mL medium.

Bioflocculant producing bacterial strain with highest flocculation activity (F.A.) was further identified on basis of physiological, biochemical, and phylogenetic characterization. Phylogenetic characterization was performed stepwise by isolating and purifying the genomic DNA (CTAB method), PCR amplification of the product and finally performing 16S rRNA gene sequencing using 27F (AGAGTTTGATCMTGGCTCAG) and 1492R (AAGTCGTAACAAGGTAACC) primers (Macrogen, Korea). The sequences obtained after 16S rRNA were assembled using FinchTV (1.4.0) and analyzed using BLAST program. Phylogenetic tree was constructed using Mega7 software. The 16S rRNA partial gene sequence was submitted to GenBank, NCBI (Batta et al., 2013).

### Optimization of Culture Conditions for Bioflocculant Production

In order to optimize the production of bioflocculant by selected bacterial strain and to enhance its flocculation efficiency; culture conditions were optimized. The effect of different carbon source *viz*. sucrose, fructose, maltose, galactose, xylose, and arabinose were studied, keeping all the other factors constant. Various nitrogen sources were examined to enhance bioflocculant production. The nitrogen sources used were urea, yeast extract, ammonium nitrate, ammonium chloride, ammonium sulfate, di-ammonium hydrogen phosphate (dAHP), and beef extract.

Initial pH of fermentation broth was adjusted from 4.0 to 11.0 (using 1N HCL and NaOH) and its effect was studied. Similarly, different inoculum concentration ranges from 1–10% was also studied. All the experiments for optimization were conducted in 250 mL Erlenmeyer flasks with 100 mL medium and the parameters for incubation were same as stated above. After optimization of all the culture conditions, fermentation study was conducted in 2.0 L flask with 1.0 L of medium.

### Purification and Characterization of Bioflocculant

A total of 1.0 L culture broth was then subjected to centrifugation (6000 rpm, 30 min, 20°C) and cell-free supernatant was used for further study. The purification of bioflocculant was performed using ethanol precipitation method as reported earlier by Subudhi et al. (2014). The precipitate obtained was dissolved in Milli-Q water and dialyzed overnight against Milli-Q water at 4°C and constant stirring (100 rpm). The final step involved precipitation (8000 rpm, 10 min, 20°C) of dialyzed sample, which was then lyophilized for further use. Partially purified bioflocculant was employed for characterization and flocculation performance studies. Chemical composition was assessed by estimation of total and neutral sugar content; quantified using Phenol-Sulfuric acid and di-nitro salicylic acid (DNSA) method, respectively. Glucose was used as standard control for the study (Chaplin and Kennedy, 1994). Protein content was measured using Bradford assay; with bovine serum albumin (BSA) as standard (Bradford, 1976). The molecular weight of protein was determined by SDS-PAGE; performing few variations in the method described by Sun et al. (2015). The bioflocculant was heated at 90°C for 5 min and then was allowed to run on gel. Fermentas (180 kDa) ladder was used as marker for the same.

Carbohydrate composition of bioflocculant BF-VB2 was determined by High Performance Liquid Chromatography (HPLC) coupled with refractive index (RI) detector. D-glucose, lactose, xylose, mannose, maltose, D-fructose, galactose, and sucrose were used as standards (Analytical grade). Ten milligrams of dried bioflocculant sample was hydrolyzed with 2 mL of 6 N HCl at 100°C in water bath for 4 h in a glass tube. The hydrolysate was then neutralized using Na_2_CO_3_. This was then centrifuged at 8000 rpm for 5 min and the supernatant was dissolved in HPLC-grade water. This was then filtered using 0.22 μm Millipore filter paper and 10 μl of sample was injected into HPLC (1220 Infinity, Agilent, United States). The parameters of HPLC run were as follows: 0.005 M H_2_SO_4_ was used as eluent, flow rate of 0.4 mL/min, the column (Aminex HPX-87H) temperature and pressure were maintained at 60°C and 30 bars, respectively.

FT-IR spectrum was recorded in spectrophotometer over a wave number range of 400–4000 cm^-1^ using KBr pellets (Spectrum GX, Perkin Elmer, United States). ^1^H NMR spectral analysis was recorded in ppm at 400 MHz using water as solvent and TMS as internal standard (400 MHZ, FTNMR, Avance III, Bruker). SEM imaging was performed for: (i) kaolin clay, (ii) purified bioflocculant, and (iii) kaolin clay particles agglomerated by bioflocculant (Chen et al., 2017a) (Zeiss, Germany).

Elemental analysis (CHNSO) of BF-VB2 was performed using an elemental analyzer (Perkin Elmer 2400). Ten milligrams of purified and freeze-dried powder of BF-VB2 was placed on tin cups and CHNSO mode of operation was selected.

The flocs formed, when crude bioflocculant was added to the synthetic turbid water suspension were assessed for their size using Particle size analyzer (Helos, H1004 and Sucell). Both the cumulative distribution (Q3%) and density distribution were measured at various range of particle size (0.5–875 μm). The dispersant used for the analysis was distilled water and the stirring speed was maintained at 60 rpm throughout.

### Flocculation Performance and Activity Assay

Flocculation efficiency of BF-VB2 obtained from strain *Bacillus* sp. TERI VB2 was assessed by treating turbid suspension of kaolin clay in water. Kaolin stimulated synthetic turbid wastewater was prepared by adding 4.0 g/L of kaolin clay powder in distilled water and dissolved through mechanical stirring for 24 h at 200 rpm. The pH of the solution was measured using pH probe (Mettler Toledo, Switzerland).

To assess flocculation activity, kaolin clay assay was performed with slight modification (omission of CaCl_2_) in method described by Batta et al. (2013). Initial tests were performed by adding 1 mL of cell-free supernatant to 9 mL of kaolin clay suspension, subjected to fast and slow mixing and after incubation at room temperature for 10 min; 1 mL of supernatant from 1–2 cm depth was collected and read against a distilled water blank. All the readings for absorbance and transmittance (T %) were measured at 550 nm in UV-VIS spectrophotometer (UV 2450, Shimadzu, Japan).

The effect of various factors (during kaolin clay assay) *viz*., pH (the pH of kaolin clay suspension was adjusted using 1 M HCl and NaOH to attain pH value of 4.0, 7.0, and 10.0, respectively), stirring time, sedimentation time (the kaolin clay suspension after treatment with bioflocculant was assessed for 5–60 min for F.A.), and concentration of BF-VB2 (0.1–1.0 mg/L) on efficacy of bioflocculant was also assessed.

Flocculation activity was expressed in percentage (%) value as:

(1) $$FA\;(\%)=\frac{\left(A-B\right)}{A}*100$$

wherein A is absorbance value of control at 550 nm, B is the final absorbance value at 550 nm (Batta et al., 2013). Similarly, transmittance was expressed as

(2) $$T\;(\%)=\frac{T_{1}-T_{2}}{T_{1}}*100$$

where T_1_ is the transmittance of initial kaolin suspension at 550 nm and T_2_ is final transmittance value of supernatant at 550 nm. Each sample was analyzed in triplicates and control was maintained using kaolin clay suspension without addition of bioflocculant. Turbidity of kaolin clay suspension was measured before and after treatment with bioflocculant BF-VB2.

Zeta potential of bioflocculant BF-VB2, kaolin suspension (both before and after treatment), and of textile dyeing wastewater were measured by performing appropriate dilutions of the samples (for wastewater samples eight times diluted sample were used). Thirty zeta runs were conducted for each sample; the temperature of the system was 25°C with count rate of 7.6 kcps and clear disposable zeta cell (Zetasizer version 7.11, Malvern, Malvern Inst. Ltd.). Zeta potential was assessed based on average of three values.

### Flocculation Process at Larger Volume

Once all the parameters for synthetic wastewater treatment were optimized, flocculation process was carried out at larger volumes to broaden this procedure for treating higher volumes of wastewater. The volumes for flocculation were increased progressively from 0.01 to 5.0 L. These experiments were carried out in appropriate glass bottles with 0.2 mg/L of bioflocculant BF-VB2 at room temperature.

### Efficiency Assessment of BF-VB2 in Comparison to Other Flocculants

A comparative study of purified BF-VB2 was performed with alum [KAl (SO_4_)_2_⋅12H_2_O] as chemical flocculant and lemon (*Citrus limon*) leaves polysaccharide as natural plant-based flocculant to treat synthetic turbid wastewater. For this, an equal dosage of both the flocculants was used as of BF-VB2 during optimization. Kaolin clay assay was considered as standard procedure for determining the flocculation activity of both the flocculants. Alum was used in the commercially available form, while total polysaccharides from lemon leaves extracted by following method as described by Manchanda et al. (2014) were used.

### Flocculation Treatment of Textile Dyeing Industrial Wastewater

The application of bioflocculant BF-VB2 was further assessed to treat wastewater from textile dyeing industry. The untreated textile dyeing wastewater was collected from a local industrial plant (New Delhi, India). The sample was stored at 4°C till further investigations.

The physico-chemical characterization of the sample was performed within 24–48 h of sample collection. pH; conductivity; salinity; were measured using 7 multi pH meter (Mettler Toledo, Switzerland), absorption maxima and UV_254_ of dyeing wastewater were assessed by UV-2450 spectrophotometer (Shimadzu, Japan), COD was measured using dichromate method based on colorimetric measurements, TSS; TDS; and TS were assessed using the earlier described methods, chloride ions were assessed using the Mohr’s AgNO_3_ titrimetric method (APHA, 2005).

Initially, from the concentration optimization studies (as described for kaolin clay) 0.2 mg/L of BF-VB2 was used for wastewater treatment (results not presented). However, not much difference was observed. Therefore, various concentrations, in the range of 0.5–3.0% (w/v) of BF-VB2 were used to treat wastewater from textile dyeing industry. This suspension was mixed first at 200 rpm for 10 min followed by 40 rpm for 5 min (Li et al., 2013). After 10 min incubation at room temperature, the supernatant was collected and examined for further analysis. However, decolorization activity, OD, and pH were assessed for a period of 5 days during which the suspension; along with a control was placed and agitated in a shaker at 37°C and 200 rpm. The samples were collected every 24 h; centrifuged at 10,000 rpm for 30 min to remove insoluble bioflocculant and analyzed.

The residual COD and color was determined (till 1 h, with a difference of 10 min) by calculating the removal efficiency as follows:

(3) $$\mathrm{Removal}\;\mathrm{efficiency}\;(\%)=\frac{C_{0}-C_{t}}{C_{0}}*100$$

where, C_0_ is the initial value and C_t_ is the value after the flocculant treatment (Gong et al., 2008). Kinetic model study for calculating the rate of reaction were also carried out for COD and dye color removal.

Another set of experiment was performed to assess the amount of dye which could be recovered from colored flocs; formed during decolorization process. A total of 0.1 M NaOH was used for desorption of dye from the flocs. Calculations were performed based on the absorbance of: (a). dye wastewater before treatment, and (b). dye recovered after treating colored flocs with NaOH.

### Statistical Analysis and Software

All the experiments carried out throughout the study were performed in triplicates. The data labels are average of these three independent experiments ± Standard deviation; presented by error bars in the graphs (<5% of average). Microsoft excel (Microsoft Office 16) has been used to carry out all the statistical analysis. For compound drawings ChemSketch (version 2018 1.1) was used.

## Results and Discussion

### Bacteria Identification, Growth Conditions, and Bioflocculant

Out of 26 bacterial strains obtained, 5 (selected on the basis of morphological assessment) were purified and examined for bioflocculant production; the bacterial strain which exhibited highest activity was selected for further studies. The results revealed that selected strain was gram positive, rod-shaped, and produced highly viscous colonies on agar plate after 24 h of incubation under aerobic conditions. Biochemical and physiological assessment displayed following characteristics of the selected bacterial strain: lysine, ornithine, urease, nitrate, arabinose, and sucrose (positive) while, citrate, H_2_S, phenylalanine, glucose, adonitol, lactose, and sorbitol (negative).

16S rRNA partial gene sequence (692 bp) of bacterial strain TERI VB2 was compared with the corresponding sequences of other bacterial strains available in database of GenBank, through BLAST and was submitted in GenBank database under accession number MF362685 (Supplementary Figure S1). The similarity analyzed with type strain *Bacillus* sp. was as high as 99.0%. According to the morphological, physiological, biochemical, and phylogenetic analysis the isolated strain was identified and named as *Bacillus* sp. TERI VB2.

The purified bioflocculant obtained from this bacterium was named as BF-VB2. Several strains of Bacillaceae family have been reported as bioflocculant producers. A vast number of references favoring the above statement are available, a few of which includes *B. licheniformis* X14 (Li et al., 2009), *Bacillus* sp. XF-56 (Liu et al., 2015), *B. subtilis* F9 (Giri et al., 2015), *Bacillus* sp. (Okaiyeto et al., 2016a), etc.

The production of bioflocculant BF-VB2 by *Bacillus* sp. TERI VB2 was studied with selection of optimal media parameters (Table 1). It was quite evident that out of all the sugars assessed, sucrose proved to be the best carbon source for *Bacillus* sp. TERI VB2 (Supplementary Figure S2). On the other hand, all other carbon sources proved to be less suitable for growth and bioflocculant production. Similar data have been presented by Nwodo et al. (2014). Urea was selected as the nitrogen source in production medium for further study (Supplementary Figure S2). This is in accordance with Raza et al. (2012) who have reported that organic nitrogen sources favored both; growth and EPS production by *Pseudomonas* sp. than inorganic nitrogen sources. However, opposes Xia et al. (2008) wherein organic sources were less effective in enhancing the production than inorganic sources. The carbon and nitrogen source data is in favor with that reported by Xiong et al. (2010) for *B. licheniformis* CGMCC 2876, which produced highest F.A.% with sucrose, glucose, and urea. The optimal production and growth was observed in the pH range of 7.0–9.0 and highest flocculation activity of 95.8% was reported at pH 7.0. The initial pH of production medium is known to influence various conditions *viz*. electric charge on microbial cells, redox potential, assimilation of nutrients by microbes and enzymatic reactions (Liu et al., 2014; Zhao et al., 2016). It has been identified earlier (Salehizadeh and Shojaosadati, 2001) that in the presence of excess inoculum; bioflocculant production is inhibited while small inoculum leads to an extended stagnant phase. Based on the results from this study it was affirmed that 1–2% of inoculum concentration showed similar flocculation activity of approximately 97.0%. The optimal inoculum concentration selected for bioflocculant production by strain TERI VB2 was 1% during which the lag phase is shortened. The optimized media composition displayed that the overall production cost of this bioflocculant is quite low and thus, it could be further raised for up-scaling.

**Table 1:** Effect of media parameters on bioflocculant production by *Bacillus* sp. TERI VB2.

Carbon source	Arabinose	Fructose	Galactose	Glucose	Maltose	**Sucrose**	Xylose	–	–	–
F.A. (%)	36 ± 0.59	36.3 ± 0.23	62.2 ± 0.29	34.4 ± 0.05	37.2 ± 0.61	**95.4 ± 0.5**	34.5 ± 0.32	–	–	–
Nitrogen source	Yeast extract	Peptone	Tryptone	**Urea**	dAHP	NH_4_Cl	NH_4_NO_3_	NH_4_SO_4_	–	–
F.A. (%)	75.6 ± 0.02	29 ± 0.35	92.1 ± 0.12	**96.2 ± 0.162**	94.4 ± 0.59	56.6 ± 0.13	53.3 ± 0.6	72.1 ± 0.03	–	–
pH	4.0	5.0	6.0	**7.0**	8.0	9.0	10.0	11.0	–	–
F.A. (%)	56.7 ± 0.06	62.8 ± 0.08	71.5 ± 0.3	**95.8 ± 0.2**	93.8 ± 0.13	91.6 ± 0.09	86.7 ± 0.35	73.5 ± 0.63	–	–
Inoculum concentration (%)	**1**	2	3	4	5	6	7	8	9	10
F.A. (%)	**97.5 ± 0.35**	97.3 ± 0.82	96.4 ± 0.4	93.8 ± 0.33	91.4 ± 0.13	89.8 ± 0.08	78.4 ± 0.58	72.1 ± 0.36	65.4 ± 0.5	60.7 ± 0.17


### Characterization Study of BF-VB2

#### Yield

Under optimized conditions the yield of 10.26 g/L of BF-VB2 was obtained (Supplementary Figure S3A). The yield obtained with the same media composition by *B. licheniformis* CGMCC 2876 was 2.93 g/L (Xiong et al., 2010). The characteristic properties of purified bioflocculant BF-VB2 have been depicted in Table 2.

**Table 2:** Production and characteristic properties of bioflocculant BF-VB2.

Production and properties of BF-VB2	Results
**Production**	
Source	*Bacillus* sp. TERI VB2
Purification method	Ethanol precipitation
**Properties**	
Physiological form	Solid
Color	White
Odor	Odorless
Solubility	Water
Active pH range	4.0–10.0
Thermal stability	30–100°C
Optimal dosage	0.2 mg/L
Composition	Polysaccharide
Functional groups	O-H, C-O
H/C and N/C ratio	3.46 and 0.20
Particle size	X_90_ 23.45 μm
Turbidity reduction	99.66% ± 1.0%
Proposed flocculation mechanism	Polymer bridging


#### Chemical Composition

**FIGURE 1:**
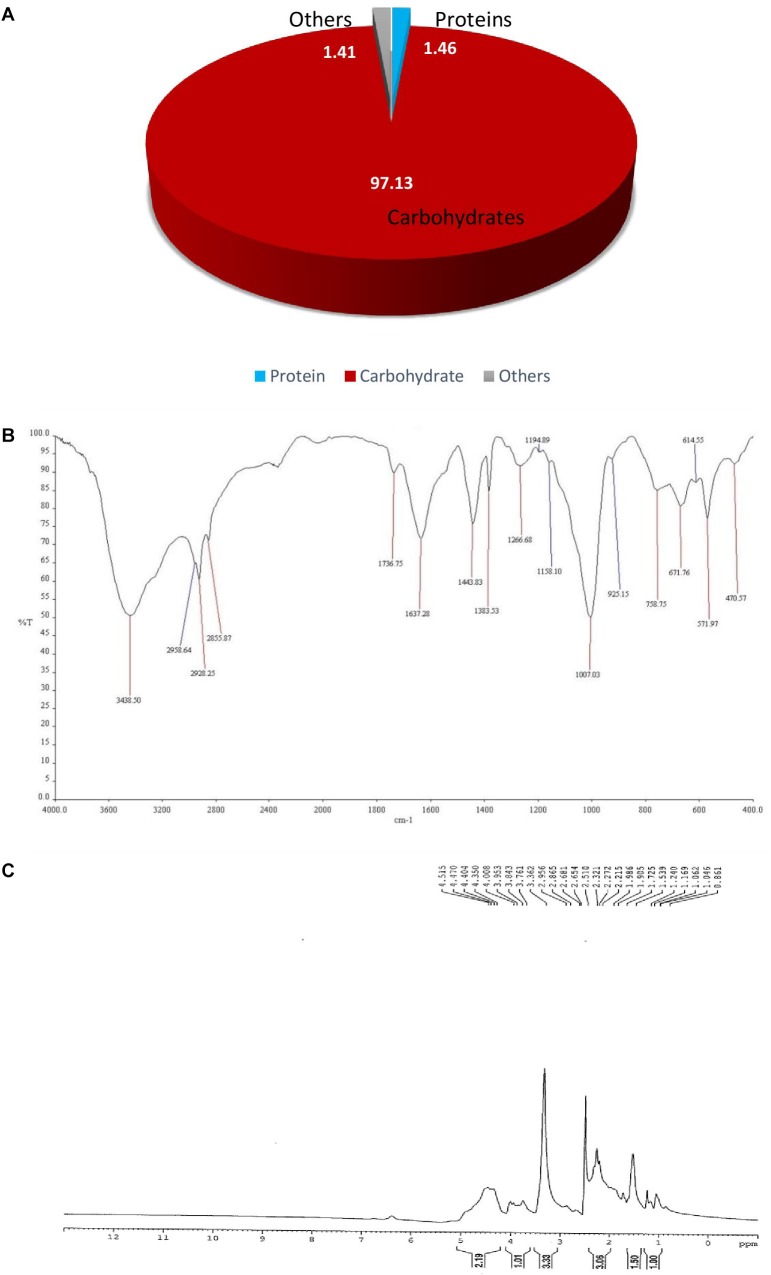
Characterization of purified bioflocculant BF-VB2. **(A)** Chemical composition; **(B)** FT-IR spectra depicting the functional groups; and **(C)** NMR spectra (^1^H).

The chemical composition of purified bioflocculant was analyzed; the total sugar and protein content of bioflocculant BF-VB2 was 97.13% ± 1.3% (64.86% ± 0.28% neutral sugars) and 1.46% ± 0.005%, respectively (Figure 1A). The results indicate that BF-VB2 is mainly composed of polysaccharides. This complies with polysaccharide bioflocculant produced by *B. licheniformis* and *Bacillus* sp. (Chen et al., 2017b; Ntozonke et al., 2017). The bioflocculant BF-VB2 was subjected to HPLC for compositional analysis. The HPLC run of bioflocculant BF-VB2 revealed the presence of glucose, fructose, sucrose, and galactose. By performing SDS-PAGE, the molecular weight of protein in the purified bioflocculant was found to be 30 kDa. Earlier, Rao et al. (2013) reported that the molecular weight of protein in bioflocculant obtained from *Bacillus amyloliquefaciens* BPRGS was 29 kDa.

#### FT-IR Spectral Analysis

The FT-IR spectra results of BF-VB2 are illustrated in Figure 1B. As presented, the spectrum of purified bioflocculant displayed a broad and strong band at 3438 cm^-1^, depicting alcohols with hydroxyl (O-H) stretch. Strong C-H bending of alkanes was observed at 2855, 2928, and 2958 cm^-1^; while, a stretch of C = O at 1736 cm^-1^ suggests presence of strong ester group. At 1383 cm^-1^ sulfate ester was also observed. In addition, absorption bands at 1266 and 1007 cm^-1^ was suggestive of amine (C-N) and strong (C-O) stretch, respectively. Interestingly, the formation of additional absorption band appeared at 1158 cm^-1^; which can be attributed to the formation of glycosidic linkages, which brings a spectral change. A medium intensity bending at 925 cm^-1^ exhibited occurrence of carboxylic acid with O-H bend. Alkyl halide (probably C-Br) peak was seen at 671 cm^-1^. The major functional groups of BF-VB2 responsible for flocculation were hydroxyl, carboxyl, amines, and halides. Occurrence of carboxyl and hydroxyl groups has been described to be effective for the flocculation process to occur; results in this study displayed the presence of these functional groups which supports BF-VB2 to be an effective flocculating agent. Further result also confirms that the main backbone of BF-VB2 is of polysaccharide.

#### ^1^H NMR Spectral Analysis

To further analyze the chemical structure, ^1^H NMR spectra of BF-VB2 were measured. As depicted in Figure 1C maximum sets of signals during ^1^H NMR was recorded in the range of 1–5 ppm. The most prominent signal at δ = 3.33 ppm was derived from the proton of ether group (HC-OR) in polymer backbone. The resonance peak at δ = 1.5 ppm is attributed to the proton of tertiary aliphatic compound (R_3_CH). At δ = 1.24 ppm a small peak depicts occurrence of proton from methylene (-CH_2_-). In addition, a triplet signal with 2n proton at a chemical shift range of δ = 2–2.5 ppm depicts HC-COOH. Also, weak signals at δ = 4.3–4.5 ppm suggests alkyl halide (C-F). According to Okaiyeto et al. (2015); carboxyl group aids in more adsorption sites for particles to attach thus, many particles can be adsorbed onto the long molecular chain of bioflocculant. The results, therefore, revealed the composition to be carbohydrate and protein substance or of glycoprotein polymeric origin. The data from ^1^H NMR in this study is in accordance with the data obtained from FT-IR.

#### Scanning Electron Microscopy Imaging

**FIGURE 2:**
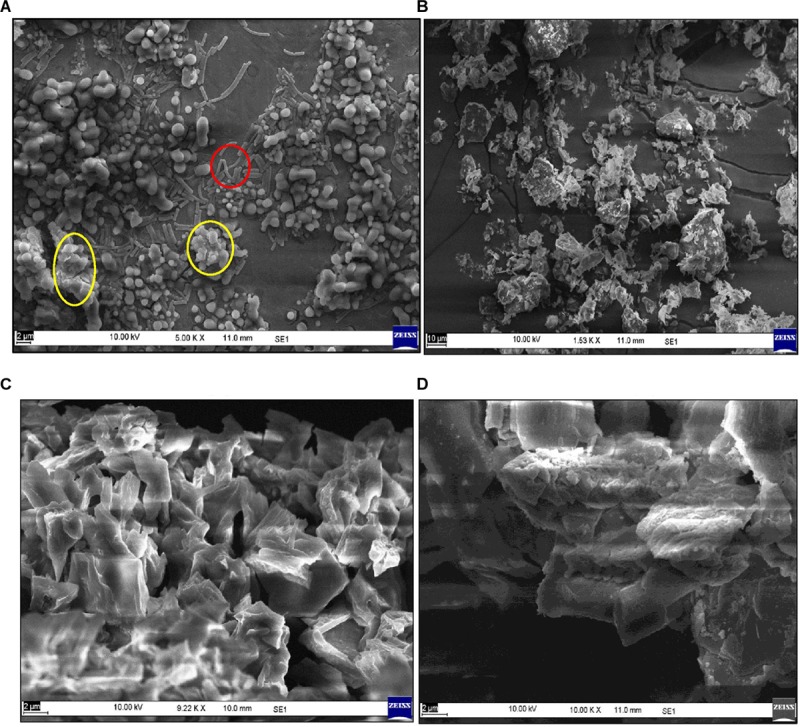
Scanning electron micrographs of **(A)** *Bacillus* sp. TERI VB2 culture broth under optimized conditions. Red color circle depicts bacteria while bioflocculant is encircled in yellow; **(B)** Kaolin clay particles; **(C)** purified bioflocculant; and **(D)** kaolin clay particles agglomerated with purified bioflocculant.

Under optimized media conditions, the strain *Bacillus* sp. TERI VB2 was raised for bioflocculant production and analyzed through SEM (Figure 2A). Surface morphology of purified BF-VB2 and its action on kaolin clay particles in turbid wastewater was also identified using SEM. As is clear from image Figure 2B, kaolin clay particles in free form are scattered and fine on the other hand, bioflocculant BF-VB2 appeared as white flakes with free sites (Figure 2C). However, when kaolin clay is treated with BF-VB2 the dispersed particles become bound to bioflocculant and form a compact floc-like structure which can be separated easily from water (Figure 2D). When flocculation occurs; first the molecular chain of polysaccharide opens making functional groups available and then loosely scattered kaolin particles interact with them which results in visible flocs.

#### Elements Percent and Floc Size

From the elemental analysis of BF-VB2 it was observed that it had a C, H, N, and O content of 36.21, 10.53, 8.61, and 19.28%, respectively, while, no Sulfur was detected. The molar element ratio for H/C and N/C were 3.466 and 0.20, respectively.

**FIGURE 3:**
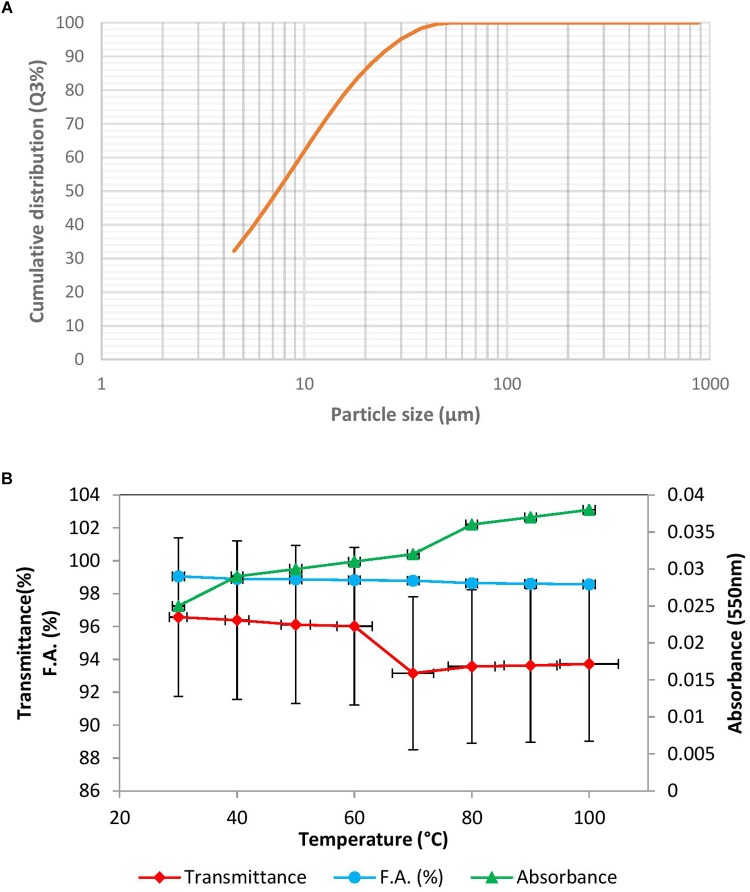
**(A)** Floc size in the presence of BF-VB2 depicted as a graph between particle size and cumulative distribution. **(B)** Thermal stability studies; effect of varying temperature on the flocculation activity of bioflocculant BF-VB2. Results are average value of triplicates and error bars represent standard deviation.

The results of floc size have been depicted as a sigmoid curve between cumulative distribution and particle size (Figure 3A). In this study X_90_ was used to indicate the floc size, which was found to be 23.45 μm. The floc formed might be described as intermediate flocs (ranging between 20 and 120 μm), as described by Lapointe and Barbeau (2017). Floc size is likely due to aggregation of particles induced by added bioflocculant. The density of the floc was 2.7100 g/cm^3^.

#### Physical Characteristics of BF-VB2

It was observed that BF-VB2 was highly soluble in water but insoluble in organic solvents. This is in accordance with the solubility principle which states that ‘like dissolves like’ (James, 1986). Further FT-IR analysis revealed the presence of hydroxyl groups within BF-VB2; this favors possibility of Hydrogen bonding with one or more water molecules, thus enhancing solubility. In contrast, BF-VB2 was insoluble in organic solvents which could again be attributed to hydroxyl groups as they add crystallinity to polymer which organic solvents are not able to break (Kumar et al., 2004).

Further, thermal stability of BF-VB2 was assessed at various temperature ranges from 30–100°C. The results are presented in Figure 3B. The results indicated that after being heated at different temperatures for 30 min, there was not much difference in the flocculation activity of BF-VB2. The flocculation activity ranged from 99.0% ± 0.5% to 98.5% ± 0.03% at lower to higher temperature range and thus, the reduction at high temperature (100°C) was just by nearly 1.0%. This can be attributed to polysaccharide chain present as the main backbone in bioflocculant. Release of extra and inner cell substances due to heating at higher temperature has been reported to be responsible for stability in flocculation activity of bioflocculant at extreme temperature range; by Gong et al. (2008). Thermal stability of BF-VB2, therefore, could initiate opportunities for practical use in the industry.

#### Flocculation Properties and Kinetics of BF-VB2

BF-VB2 was assessed for its F.A. in presence of 1.0 mL of 1.0 M various cations *viz*. Na^+^, K^+^, Li^+^, Ca^+2^, Mg^+2^, Al^+3^, and Fe^+3^ (Supplementary Figure S4). It was observed that cation addition had no positive effect on the F.A. (%) of BF-VB2. Addition of Na^+^, K^+^, Li^+^, Ca^+2^, and Mg^+2^ to kaolin stimulated wastewater have no impact on F.A. of bioflocculant BF-VB2; however, it was influenced by presence of Al^+3^ and Fe^+3^. The results thus, indicated that BF-VB2 is a cation-independent bioflocculant. Similar results have been depicted in bioflocculant produced by *Enterobacter* sp. ETH-2 (Tang et al., 2014). The cation-independence nature adds up to a positive point toward application of BF-VB2 as it has been stated by Wan et al. (2013) that; for a bacterial bioflocculant to possess high flocculation efficiency without any external cationic source is a desirable property as it assists to save cost and reduce risk of contamination. Although, it has been reported that for enhancing the F.A. of cation-dependent flocculants, addition of metal ions plays a vital role (Sun et al., 2015).

#### Effect of pH

**FIGURE 4:**
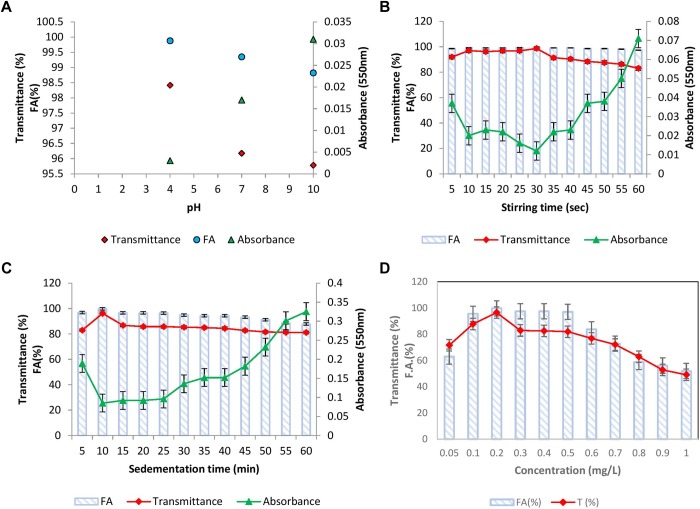
Flocculation properties of bioflocculant BF-VB2: **(A)** effect of kaolin clay suspension pH; **(B)** effect of stirring time; **(C)** effect of sedimentation time; and **(D)** effect of bioflocculant BF-VB2 dosage. All data points are mean of triplicates ± SD.

The effect of pH of kaolin clay suspension on flocculation activity was examined and the results have been presented in Figure 4A. It was noticed that at all the pH tested, BF-VB2 displayed a slight variation in flocculation activity and transmittance. The highest activity and transmittance was observed at pH 4.0 of 99.8% ± 0.2% and 98.4% ± 0.5% followed by 99.3% ± 0.23% and 96.1% ± 0.6% at pH 7.0 and 98.8% ± 0.02% and 95.7% ± 0.05% at pH 10.0, respectively. Gong et al. (2008) stated that optimal pH value for polysaccharide bioflocculant SF-1 was in the range of 5.0–7.0. Sun et al. (2015) on the other hand reported highest flocculation activity at pH 11.0. Although it has been recorded earlier that many bacterial bioflocculant works effectively at wide range of pH, still the present study could be considered distinctive from others due to stability in F.A. and T (%) in all pH range. The results thus render cost-effective (as there is no need to adjust pH) role of BF-VB2 in treatment of wastewater with varying pH. Further, at low pH (4.0) self-aggregation of kaolin particles due to protonation effect was also observed which might have contributed toward higher activity. Therefore, for other experimental work, pH 7.0 was considered as optimal pH for kaolin suspension.

#### Effect of Stirring Time

To bring about the interaction of kaolin particles and flocculant added, flocculation assay involves two steps of mixing. First, rapid stirring distributes the flocculant uniformly in water followed by slow stirring which stimulate contact and collision of flocculants and kaolin particles (Ma et al., 2017). This assists floc formation and thus serves to be a crucial step to consider during flocculation. Therefore, stirring speed and time during slow mixing were investigated. The stirring speed was kept constant at 40 rpm and the effect of variation in time of stirring was assessed. Figure 4B depicts that till 10–40 s of stirring, F.A. was maintained at nearly 99.0% ± 0.5% while T (%) of supernatant was maximum (nearly 98.0% ± 0.3%) at 30 s. It was observed that the value of transmittance begun to reduce with further increase in stirring time, therefore, 30 s slow mixing was kept constant for further experiments. It has been established earlier that as the stirring time is increased further from the optimum value required for collision of particles, flocs tend to break easily into smaller particles (Ma et al., 2017).

#### Effect of Sedimentation Time

To examine the settling property of flocs, sedimentation time (5–60 min) was analyzed by measuring transmittance and absorbance. The results depicted in Figure 4C showed that the rate of sedimentation of kaolin clay particles by BF-VB2 is quite decent. The transmittance value achieved in the initial 5 min was 82.8% ± 0.18% with a F.A. of 96.8% ± 0.36%. This suggests that in a short span of time sedimentation of most flocs have started. The highest value of 96.1% ± 0.26% and 99.7% ± 0.35% transmittance and F.A., respectively, was obtained at 10 min. On increasing the time of sedimentation further till 60 min; results obtained fluctuated between 86.0–81.0%. Similar trend in results for T (%) has been reported by Ma et al. (2017) with cationic polyacrylamide (PAMC).

In addition to F.A. (%) the transmittance was also recorded for pH, stirring and sedimentation time study. The measurement of transmittance (%) is an indicator of water quality, it gives the percentage of light which could pass a given distance through water (1 cm path length was used in this study). The transmittance values obtained during the flocculation of kaolin clay stimulated wastewater throughout was more then 95.0% and therefore, it proves that the quality of synthetic wastewater was improved at par by bioflocculant BF-VB2. So far, no reports are available on transmittance study of synthetic wastewater using bacterial flocculant, however, Ma et al. (2017) have reported in their study the transmittance by cationic polyacrylamide flocculation.

#### Effect of Bioflocculant Concentration

The effect of bioflocculant concentration at ranges from 0.1–1.0 mg/L, respectively; with 0.1 difference was studied (Figure 4D). Flocculation activity of over 95.0% ± 1.5% was reported in the range of 0.1–0.5 mg/L and the maximum F.A. of 99.0% ± 0.5% was observed at an optimum dosage of 0.2 mg/L. This optimal dosage value was then employed to calculate maximum adsorption capacity of BF-VB2 (Supplementary Equation S1). The results indicated that 1980.0 mg ± 5.0 mg of kaolin particles can be adsorbed by 0.2 mg of BF-VB2 in less than 10 min. Beyond 0.5 mg/L, reduced F.A. maybe precisely due to re-stabilization caused by change in kaolin particle charge at higher concentration of bioflocculant. On the other hand, at insufficient concentration of bioflocculant, bridging is not formed effectively (Gong et al., 2008). The reduction in turbidity assessed before and after treatment with BF-VB2 was significant. The initial turbidity of synthetic turbid water suspension measured was 1360.0 NTU ± 2.8 NTU, which was reduced to 4.6 NTU ± 0.05 NTU. The stages of floc formation by BF-VB2 have been shown in Supplementary Figure S3B.

#### Flocculation Mechanism for Synthetic Contaminant

The average zeta potential value of bioflocculant BF-VB2 (at highest flocculation activity) was -19.8 mV ± 2.8 mV. Based on the value the charge on bioflocculant was found to be negative. Similarly, the zeta potential on kaolin clay suspension before treatment was also negative (-26.7 mV ±7.87 mV) at pH 8.0. In addition, zeta potential of kaolin clay suspension after treatment with BF-VB2 was slightly increased to -23.6 mV ± 5.01 mV (Supplementary Figure S5). It has been reported earlier that greater the zeta potential more stable the suspension is (Lopez-Maldonado et al., 2014). The result quality in all the three runs was found to be good.

**FIGURE 5:**
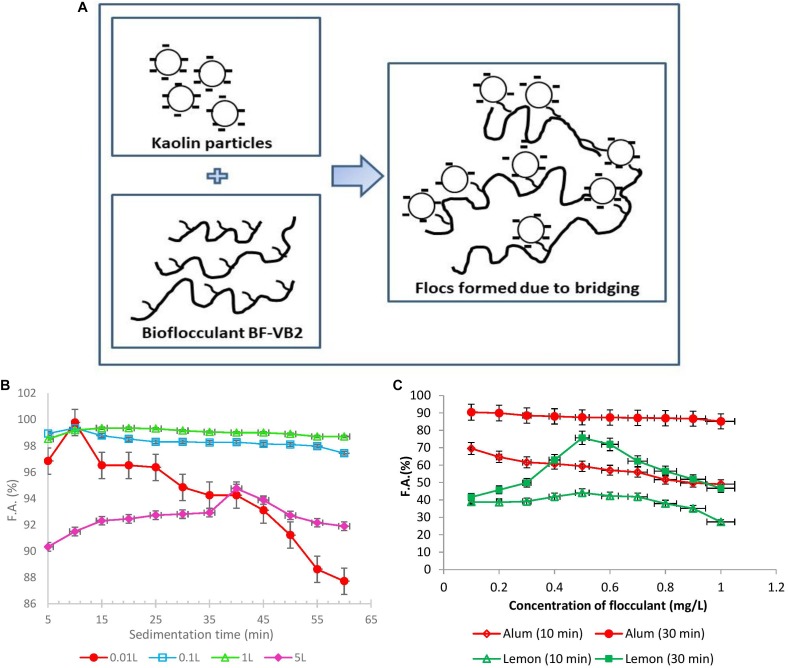
**(A)** Schematic I. Proposed flocculation mechanism of bridging of kaolin suspension with bioflocculant BF-VB2. **(B)** Graphical representation of F.A. (%) with sedimentation time *wrt* large volumes of flocculation of wastewater. **(C)** Effect of dosage of alum as chemical and lemon polysaccharide on flocculation activity of turbid wastewater stimulated with kaolin clay after different incubation time (10 and 30 min). The values of data points imply average of three independent experiments ± SD.

From all the observations during this study and by zeta potential analysis, the most probable mechanism which could define flocculation by bioflocculant BF-VB2 is polymer bridging Schematic I (Figure 5A). Bridging mechanism involves formation of a three-dimensional complex structure in which polysaccharide bioflocculant acts as a bridging element between two kaolin particles (kaolin particle-polymer-kaolin particle). This can be attributed to the presence of hydroxyl groups in bioflocculant BF-VB2, as analyzed by FT-IR study. In the presence of hydroxyl groups in bioflocculant BF-VB2: (i) interaction between bioflocculant and kaolin clay particles occur due to ionic bonding and (ii) H-bonding aids bioflocculant to be adsorbed on to the surface of kaolin particles. This ultimately leads to extension of functional moieties in BF-VB2 beyond the surface of particles into the solution which then attracts other particles with vacant adsorption sites (Lee et al., 2014). In addition, polysaccharide backbone with active sites may also be considered as one of the factors for high flocculation activity of BF-VB2. Bridging have also been proposed earlier for bioflocculant from *Enterobacter* sp. ETH-2 and MBF-UFH bioflocculant by Tang et al. (2014) and Okaiyeto et al. (2015).

#### Flocculation at Larger Volume and Shelf Life Study

As a step forward for treatment of wastewater at a higher scale, flocculation using BF-VB2 was performed at larger volumes. The process was conducted from 0.01, 0.1 to 5.0 L. After conducting the flocculation experiments the wastewater were evaluated for F.A. (%) with time and compared for increase in the F.A. (%) (Figure 5B). During treating wastewater at 0.01 L in test tubes, it was noted that with further increase in time after 10 min there was a drop in the F.A. (%). However, when the wastewater volume was increased from 0.01 to 0.1 and 1.0 L (conducted in glass bottles) a stability in F.A. (%) was observed. The maximum F.A. observed at 5.0 L was 94.76% ± 2.89%. The minimum F.A. (%) in 0.1; 1.0 L; and 5.0 L were 97.44% ± 1.99%; 98.71% ± 0.38%; and 90.32% ± 0.08%, respectively. Hence, it could be concluded that a linear and stable trend was maintained at higher volumes. Further, this also reveals that the flocs formed during flocculation process by BF-VB2 were strong and stable.

It has been stated that shorter shelf-life restricts natural polymers and therefore, to develop flocculants; with longer shelf-life is important (Lee et al., 2014). Hence, shelf-life of BF-VB2 was examined for 6 months and it was observed that there was not much reduction in the F.A. (%) of BF-VB2 over time. Even after 6 months of incubation at room temperature, the purified bioflocculant maintained a F.A. (%) of 97.89% ± 2.65%. To the best of our knowledge there are no reports available for shelf-life study of a bacterial bioflocculant and stability has only been described in the form of pH and thermal study. However, it has been reported that shelf-life of liquid flocculants is generally 3–6 months and for emulsions is about a year^3^. In this study we have concluded that activity of BF-VB2 (in powdered form) was retained up to 6 months.

#### Comparison of BF-VB2 Efficiency With Other Flocculants

Efficiency of BF-VB2 to act as flocculant in turbid water treatment was compared with alum and lemon polysaccharides (Supplementary Figure S6). Further, Figure 5C presents a comparison in activity of conventional flocculant; alum and a plant-based natural flocculant; lemon polysaccharide, to flocculate turbid wastewater. The results suggest a great potential of BF-VB2 to be used in wastewater treatment along with the other flocculants. In case of alum, the maximum flocculation activity of 90.4% ± 3.88% was achieved after 30 min of incubation at room temperature at 0.1 mg/L concentration. A moderate activity in the range of nearly 80.0% ± 1.05% was retained as the concentration was further increased to 1.0 mg/L. It was also observed that after 10 min of incubation at room temperature, the flocculation activity was very low. In contrary, when lemon polysaccharide was used, the maximum flocculation activity of 75.68% ± 0.80% was reported after 30 min of incubation. However, the highest reported flocculation activity value for BF-VB2 in this study was 99.0% ± 0.5%. Although the effective concentration of alum was low as compared to BF-VB2, the later avoids environmental issues. The findings from this study thus revealed that BF-VB2 was significantly more efficient than other examined flocculants in treating synthetic turbid wastewater stimulated with kaolin clay. To the best of our knowledge, lemon leaves polysaccharide has not been reported earlier for both; (a) flocculation efficiency, and (b) comparative analysis with other flocculants.

### Textile Dyeing Wastewater Treatment Using BF-VB2

#### Characterization of Sample

Present study confirmed efficiency of bioflocculant BF-VB2 to possess high flocculation activity without any additional cation and at a wide pH range; to treat synthetic wastewater. Therefore, to further characterize activity of BF-VB2, it was subjected to treat wastewater from textile dyeing industry. The sample was characterized (as listed in Table 3), it had a pH value of 9.15 with presence of chloride ions.

**Table 3:** Physico-chemical characterization of wastewater sample from textile dyeing industry.

Parameters	Textile dyeing wastewater
Color	Dark blue
Odor	Objectionable
pH	9.15
Absorbance (λ_max_ 620 nm)	2.452
Turbidity (NTU)	11.4
UV_254nm_	0.587
COD (mg/L)	2,400 ± 9.03
TDS (mg/L)	9,860 ± 5.0
TSS (mg/L)	13,200 ± 0.98
TS (mg/L)	23,060 ± 5.98
Conductivity (ms/cm)	19.55 ± 0.35
Salinity (PSU)	11.73 ± 0.06
Cl^-^ (mg/L)	5798 ± 1.32


#### Flocculation Performance

**FIGURE 6:**
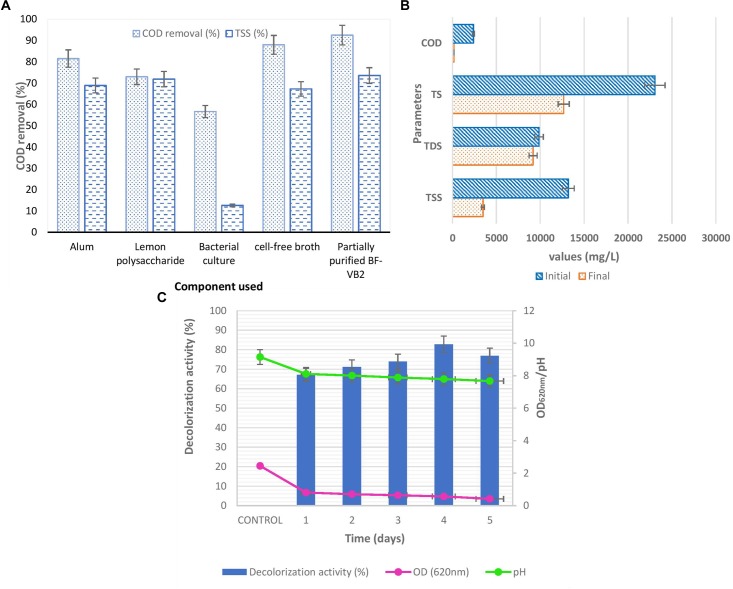
**(A)** Comparison chart of selected components for flocculation process of textile dyeing wastewater on COD and TSS removal. **(B)** Graph depicting activity of BF-VB2 in treating various parameters of textile dyeing industry wastewater. **(C)** Study of decolorization activity, pH and OD *wrt* time. Results are average value of triplicates and error bars represent standard deviation.

Various components of *Bacillus* sp. TERI VB2 (bacterial suspension; cell-free supernatant; and partially purified BF-VB2), along with other flocculants (alum and lemon polysaccharide) were evaluated for their efficiency in treating textile dyeing industry wastewater. It was observed that out of all the 5 components used, partially purified BF-VB2 depicted maximum reduction in COD and TSS (Figure 6A). This illustrates the potential activity of BF-VB2.

The reduction in COD and TSS were 92.54% ± 0.24%, and 73.59% ± 0.71%; while, it was observed that the decrease in TDS was only 6.86% ± 0.006% and thus, the overall reduction in TS was 45.06% ± 0.71% (Figure 6B). Although, not much difference in conductivity and salinity were observed; which is clear from the values obtained for TDS reduction. Further, after flocculation the color of textile dyeing industry wastewater turned from dark blue to light blue. The decolorization of dye from wastewater sample was analyzed by monitoring the reduction in absorbance peak at the absorption maxima of the dye. It was observed from Figure 6C, that reduction in dye color was 82.78% ± 3.03% on fourth day of incubation with a drop in pH of 7.79 ± 0.12. The absorbance value at wavelength maxima, i.e., 620 nm decreased to 0.422. The value of chloride ions was dropped to 1049.0 mg/L ± 2.6 mg/L after treatment, i.e., 81.90% ± 2.0% reduction.

As discussed above, during the flocculation process about 82.0% of the color present in wastewater was transferred to flocs. This color was then desorbed from flocs using NaOH and it was observed that use of alkaline solution directed to nearly 65.0% recovery of dye from flocs. The ability of alkaline solutions to reduce the dye affinity toward flocculants have been established earlier (Szygula et al., 2009). If these flocs could be reused, then this may prove to be energy and cost-efficient process.

#### Kinetic Model and Mechanism

**FIGURE 7:**
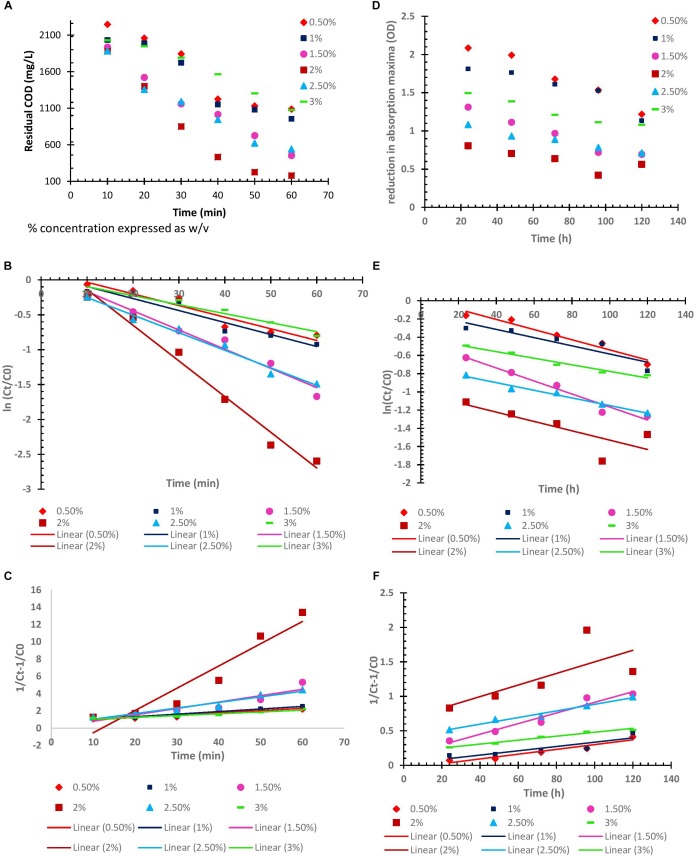
**(A–C)** Zero, pseudo first, and pseudo second order kinetics of flocculation process (in terms of COD reduction). **(D–F)** Zero, pseudo first, and pseudo second order kinetics of flocculation process (in terms of decolorization), using different concentrations of BF-VB2 *wrt* time.

In order to study the kinetics for degradation of organic contaminants (in terms of COD), and decolorization of dye; by BF-VB2, both; type and rate of reaction were examined. The study was carried out at room temperature without changing the pH value of wastewater. However, concentration of BF-VB2 was varied from 0.5 to 3.0% (w/v). For zero order, pseudo-first order, and pseudo-second order kinetic model’s, graphs were first plotted as: C_t_ vs. t (Figure 7A,D), ln (C_t_/C0) (Figure 7B,E), and 1/C_t_-1/C0 (Figure 7C,F), respectively. The results illustrated that decrease in both, COD, and dye color occurred with increase in time. From the graphs the regression coefficient and rate of reaction (k) (or slope) were calculated (as shown in Table 4).

**Table 4:** Pseudo first order kinetics corresponding k (rate of reaction) and R^2^ values (from graph) for different concentration of BF-VB2 (A) for COD reduction, and (B) for dye decolorization.

Dose of BF-VB2 (%) w/v	R^2^ (from graph)	K (from graph)	K (calculated)
	**A**

0.5	0.92	-0.0167	1.31 × 10^-2^
1.0	0.92	-0.0172	1.5 × 10^-2^
1.5	0.97	-0.027	2.7 × 10^-2^
2.0	0.98	-0.0511	4.32 × 10^-2^
2.5	0.98	-0.0252	2.4 × 10^-2^
3.0	0.94	-0.0129	1.35 × 10^-2^
R^2^ and k represents regression and slope from the graph			

	**B**

0.5	0.95	-0.0056	5.8 × 10^-3^
1.0	0.83	-0.0045	6.4 × 10^-3^
1.5	0.96	-0.0071	1.05 × 10^-2^
2.0	0.62	-0.0051	1.83 × 10^-2^
2.5	0.98	-0.0042	1.02 × 10^-2^
3.0	0.96	-0.0036	6.8 × 10^-3^
R^2^ and k represents regression and slope from the graph			


Figure 7A,D depicts effect of different concentration [0.5% to 3.0% (w/v)] of bioflocculant BF-VB2 on COD and dye color removal, respectively, with respect to contact time. This represents the zero-order reaction kinetics. The results from the same illustrated that with increase in the concentration of BF-VB2 from 0.5 to 2.0% (w/v), COD and dye color reduction increased significantly. However, further increase in the concentration of BF-VB2, i.e., 2.5 and 3.0% (w/v) decreased the removal rate. The maximum reduction rates were observed with 2.0% (w/v) and hence, it was selected as the optimum concentration of BF-VB2 required to treat these impurities in the textile dyeing wastewater sample. This suggests that 2.0% concentration is possibly enough to create the active sites for degradation.

Further, from the graphs for pseudo first and second order kinetics it was concluded that the flocculation of textile dyeing wastewater by bioflocculant BF-VB2 for COD and dye color removal best fitted pseudo-first order kinetic model. This was suggestive from the regression coefficient (R^2^) values from the graphs which was >0.9 for all the different concentrations of BF-VB2 studied. The results of this study for COD removal kinetics agrees with that of Jimenez et al. (2005) who have defined the removal of particulate COD in activated sludge process; however, according to Karthikeyan et al. (2015) COD removal for tannery dyeing wastewater followed pseudo-second order kinetics. The values for pseudo-second order reaction are also presented in Supplementary Table S1. The rate constant values were calculated as follows:

(4) $$C_{t}=C_{0}.e^{-kt}$$

(5) $$\ln\;C_{0}/C_{t}=k_{1}t$$

(6) $$\frac{1}{C_{t}}-\frac{1}{C_{0}}=k_{2}t$$

Where, C_0_ and C_t_ are same as stated earlier for eq. (3) and k, k_1_, and k_2_ are rate constants for zero, pseudo first and second order reactions.

It was observed that the highest kinetic rate constant for COD and dye removal were: 4.3 × 10^-2^ and 1.83 × 10^-2^, respectively, at BF-VB2 dosage of 2.0% (w/v). This could be attributed to the active sites which are available and responsible for catalytic degradation of compounds under study (Karthikeyan et al., 2015). Therefore, the optimal concentration of BF-VB2 for treatment of textile dyeing wastewater is 2.0% (w/v). However, the rate of reaction for removal of dye color was slow in comparison to COD removal.

**FIGURE 8:**
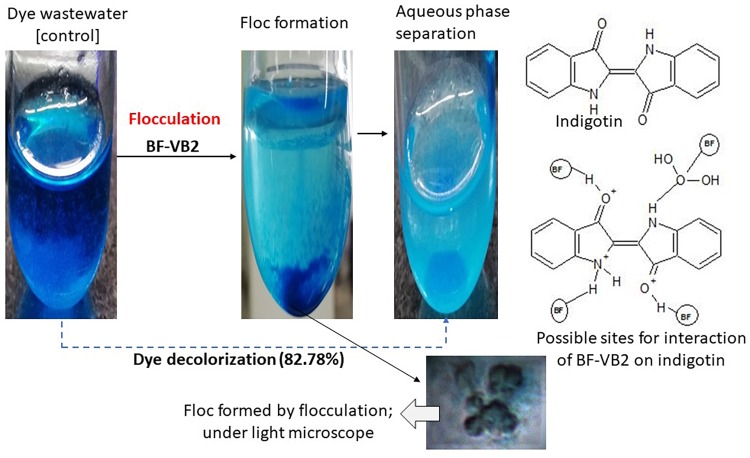
Schematic illustration of BF-VB2 in aqueous solution and possible dominant molecular interaction associated with adsorption and bridging of dye molecules.

As stated in the previous section, bridging was proposed as the mechanism of action for BF-VB2 on synthetic contaminant (kaolin clay). Similarly, the efficiency of BF-VB2 in treating real wastewater samples could also be suggestive of the various functional groups present. The most probable methods described best as mechanism for flocculation involves bridging, charge neutralization, or a combination of the two. Charge neutralization is applicable for flocculation only when the charge on both, i.e., particles in suspension and bioflocculant are opposite in nature (Lee et al., 2014). However, in the present study it was observed that wastewater sample and bioflocculant had same charge (negative) on their surface. The charge on the surface of textile dyeing wastewater was -51.3 mV ± 6.085 mV (average of triplicates of eight times diluted sample). It could also be concluded that floc formation in presence of BF-VB2 takes place by both hydrophilic as well as hydrophobic interactions. Amine and hydroxyl groups interacts with the negatively charged surfaces. Alcohols and amines may possibly be the reason for H-bridging while, -CH and -CH_2_ could be responsible for the hydrophobic interactions. This mechanism thus is also responsible in reducing the levels of chloride ions from wastewater samples. From the study it was observed that dye molecules present in textile dyeing wastewater sample were of indigotin (based on absorption maxima of the dye) and the probable sites of possible interactions has been depicted in Schematic II (Figure 8). Similar studies (for bridging) have been made for several bacterial bioflocculants and there are also reports available on charge neutralization as the probable mechanism of flocculation; few of which have been combined in Table 5.

**Table 5:** A comparative analysis of flocculation mechanism exhibited by various bacterial flocculants.

Bacteria/bioflocculant	Mechanism of action	Reference
*Proteus mirabilis* TJ-1	Bridging	Xia et al., 2008
*Bacillus megaterium* TF10	Bridging	Yuan et al., 2011
*Enterobacter* sp. ETH-2	Bridging	Tang et al., 2014
EPS-1	Charge neutralization	Sun et al., 2015
*Arthrobacter humicola*	Charge neutralization	Agunbiade et al., 2017
*Bacillus* sp. TERI-VB2	Bridging	This study


These results suggest feasibility of bioflocculant BF-VB2 in wastewater treatment for dyeing industries. Further, extensive research on the characterization of wastewater before and after treatment with bioflocculant BF-VB2 is in progress. The future study would further address the mechanism of action of the bacterial bioflocculant BF-VB2 on industrial wastewater from different sources.

## Conclusion

A promising novel bioflocculant BF-VB2 (10.26 g/L) was produced from *Bacillus* sp. TERI VB2 with carbon and nitrogen source which aided in low production cost. Characteristic features, *viz*., high flocculation efficiency, turbidity removal, transmittance, thermal and pH stability, and cation independence suggest that it is capable enough to be mentioned as a potential water cleaner. Low dosage, faster sedimentation, and enhanced shelf-life further favor the above statement. A comparative study between flocculation process revealed bacterial flocculant to be more efficient.

Progressive process of flocculation at large volumes produced substantial results. This research thus provides helpful insights into development of an upscale wastewater flocculation process using effective flocculating agent (BF-VB2); which could be produced at higher level and commercialized. However, further scale-up of the flocculation process still demands for investigation.

## Data Availability

The datasets generated for this study can be found in GenBank, MF362685.

## Author Contributions

VB prepared the basic research outline, conducted all the laboratory experiments and data analysis, and wrote the manuscript. BL supported in planning, writing, reviewing, and revising the manuscript.

## Conflict of Interest Statement

The authors declare that the research was conducted in the absence of any commercial or financial relationships that could be construed as a potential conflict of interest.

## Footnotes

1 https://www.siwi.org/wp-content/uploads/2017/06/Water-Governance-Mapping-Report-INDIA.pdf

2 https://www.academia.edu/8537218/TEXTILE_DYEING_INDUSTRIES_IN_INDIA (accessed March 20, 2019).

3 http://tramfloc.com/flocculants/ (accessed April 24, 2019).
