# Supplementary material for: Exploration of Performance Kinetics and Mechanism of Action of a Potential Novel Bioflocculant BF-VB2 on Clay and Dye Wastewater Flocculation
Source: Front Microbiol. 2019 Jun 7;10:1288. doi: 10.3389/fmicb.2019.01288 (PMC6568053; doi:10.3389/fmicb.2019.01288)
Supplement: Supplementary file 1 [file Data_Sheet_1.PDF]

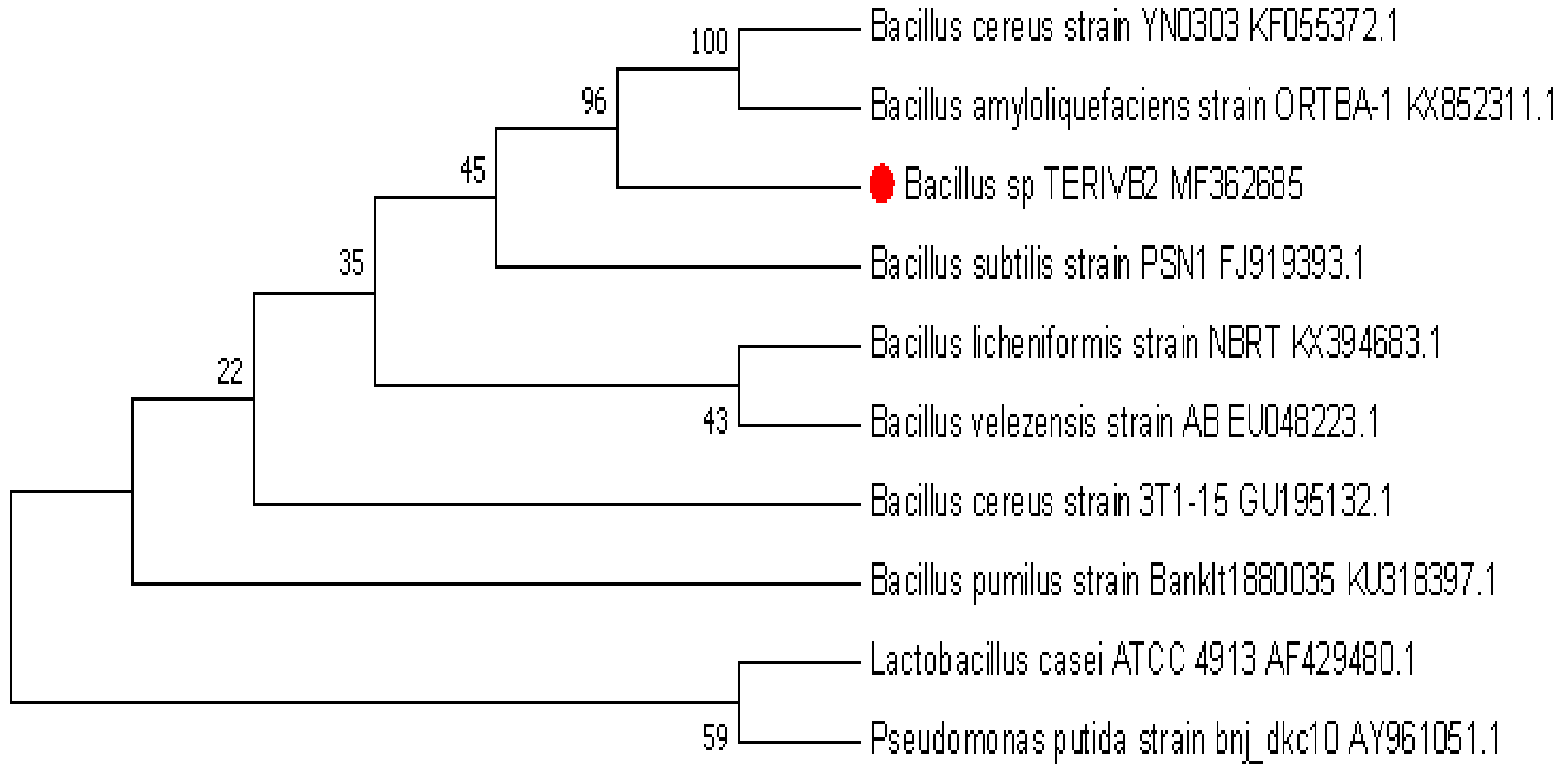

Figure S1. Neighbour-joining tree depicting the Phylogenetic position of bioflocculant BF-VB2 producing bacterial isolate *Bacillus* sp. TERI VB2, based on 16S rRNA gene sequence. Accession numbers are also provided.

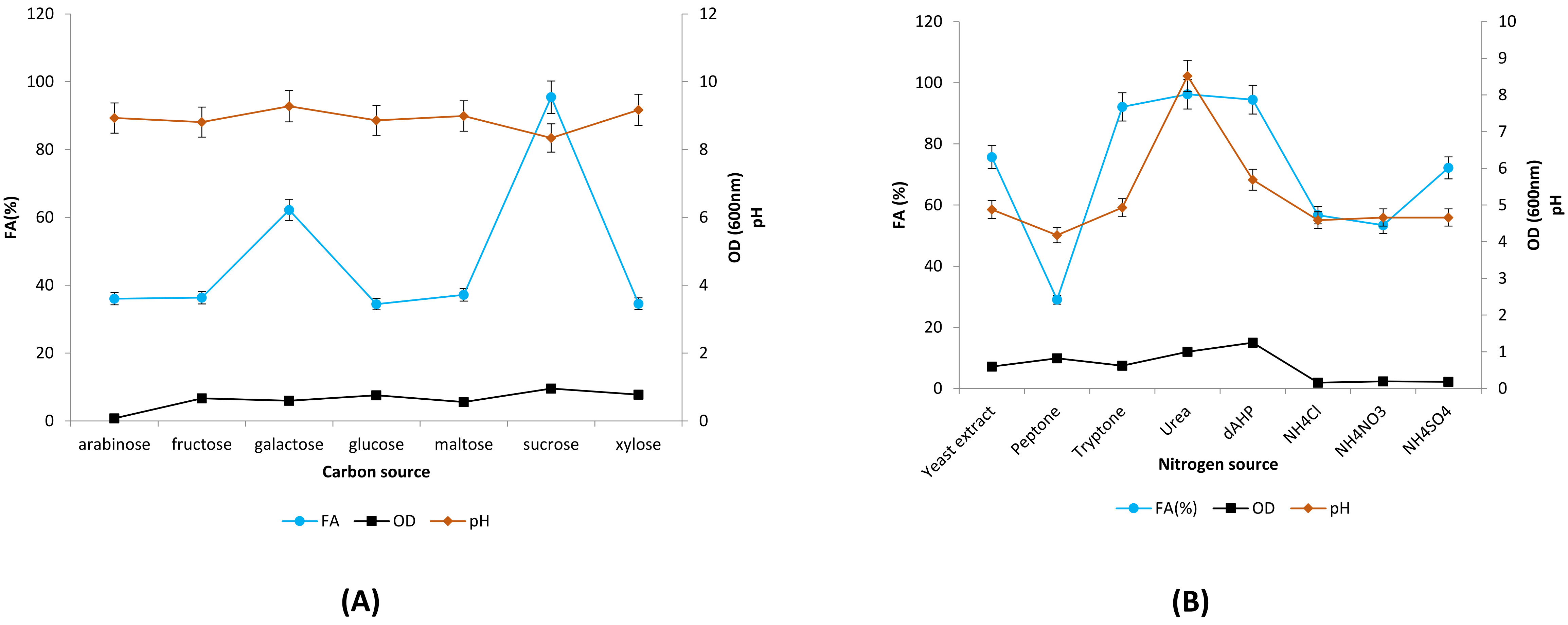

Figure S2. Optimization studies (A). effect of various carbon sources on flocculation activity and (B). effect of various nitrogen sources on flocculation activity.

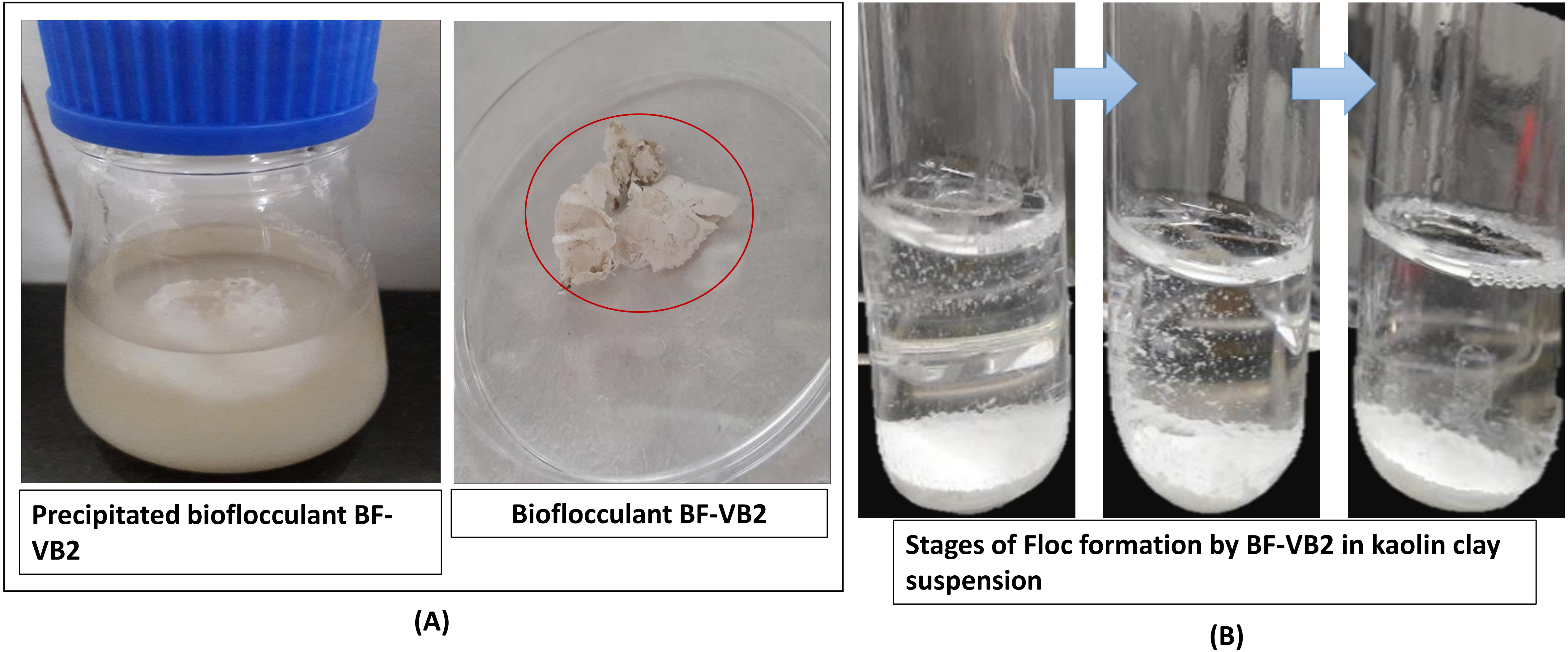

Figure S3. (A). Bioflocculant BF-VB2 produced by *Bacillus* sp. TERI VB2; both in precipitated and lyophilized form; (B). Formation of flocs after flocculation of synthetic turbid wastewater by BF-VB2.

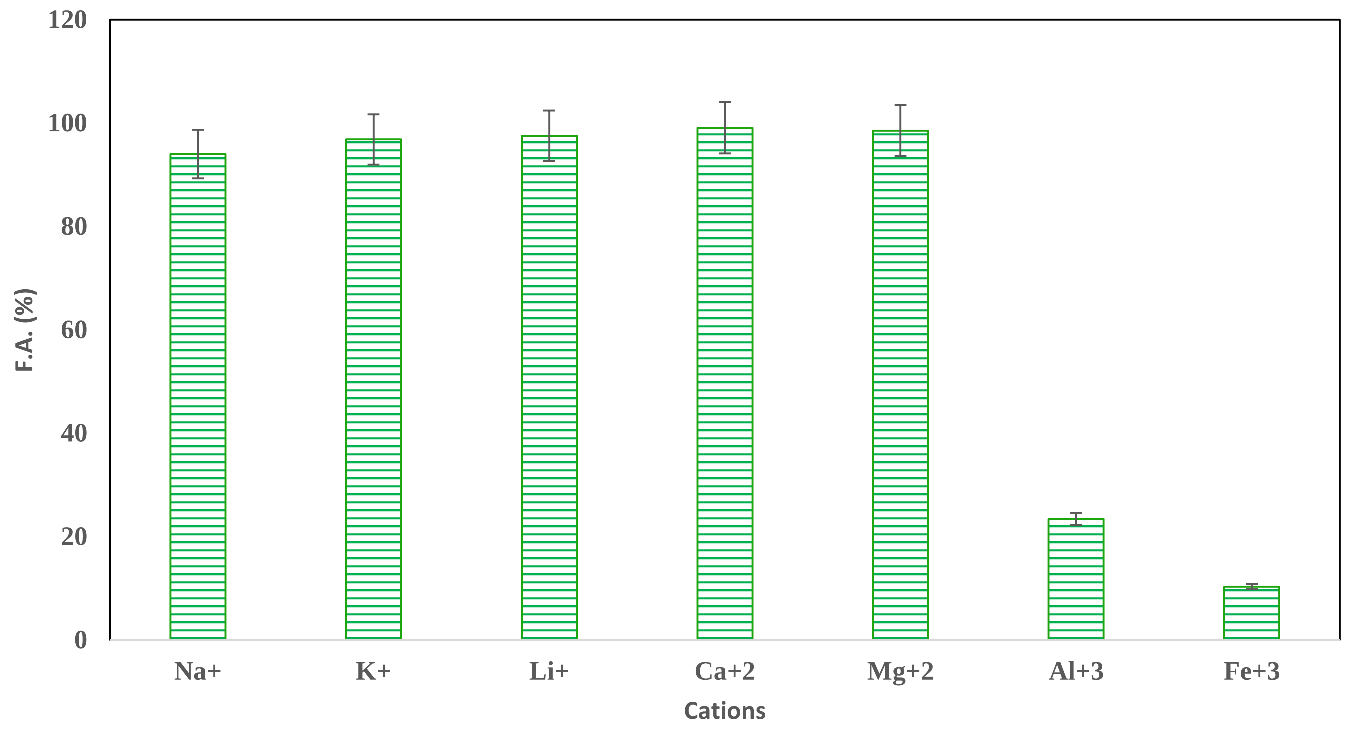

Figure S4. Effect of different cations on the flocculation activity (F.A.%) of bioflocculant BF-VB2 for kaolin stimulated wastewater

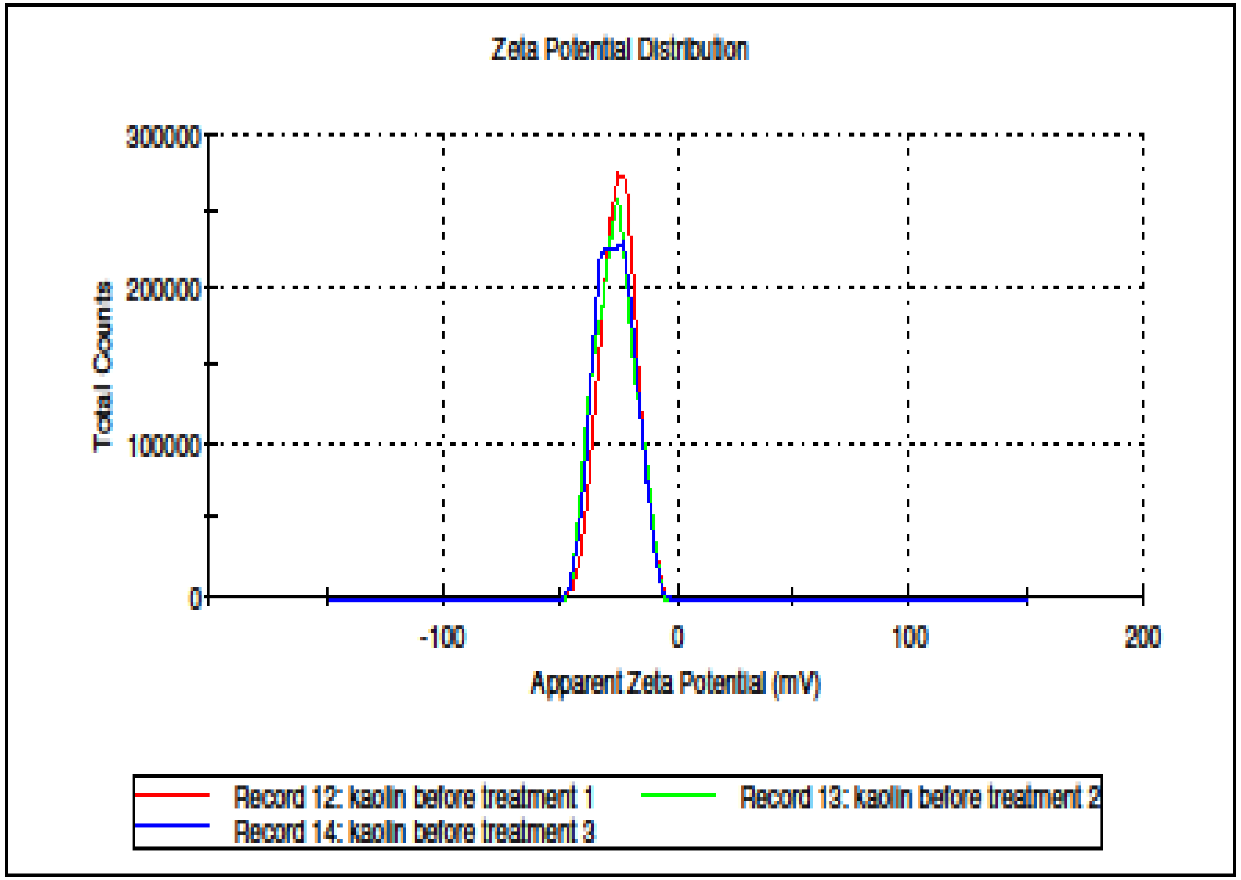

(A)

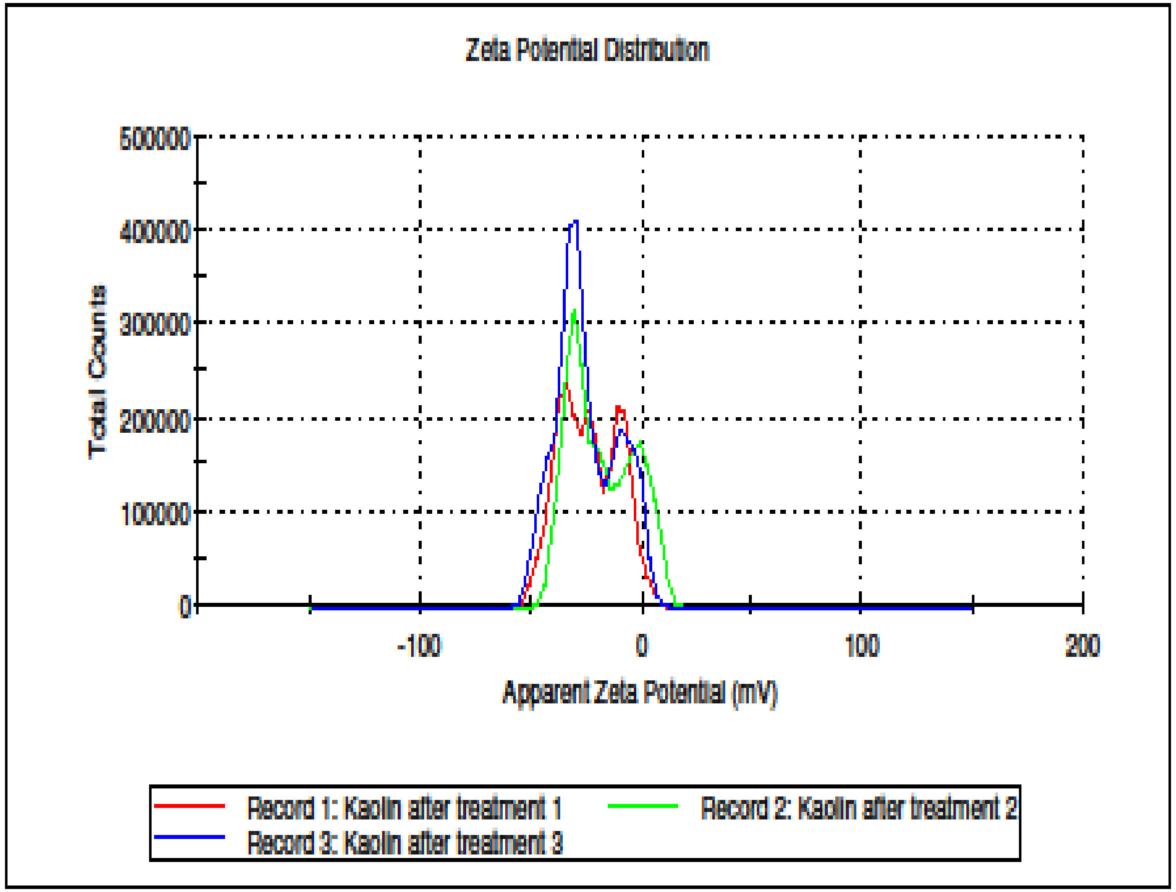

(B)

Figure S5. Zeta potential distribution of kaolin clay suspension (A). Before treatment and (B). After treatment with BF-VB2.

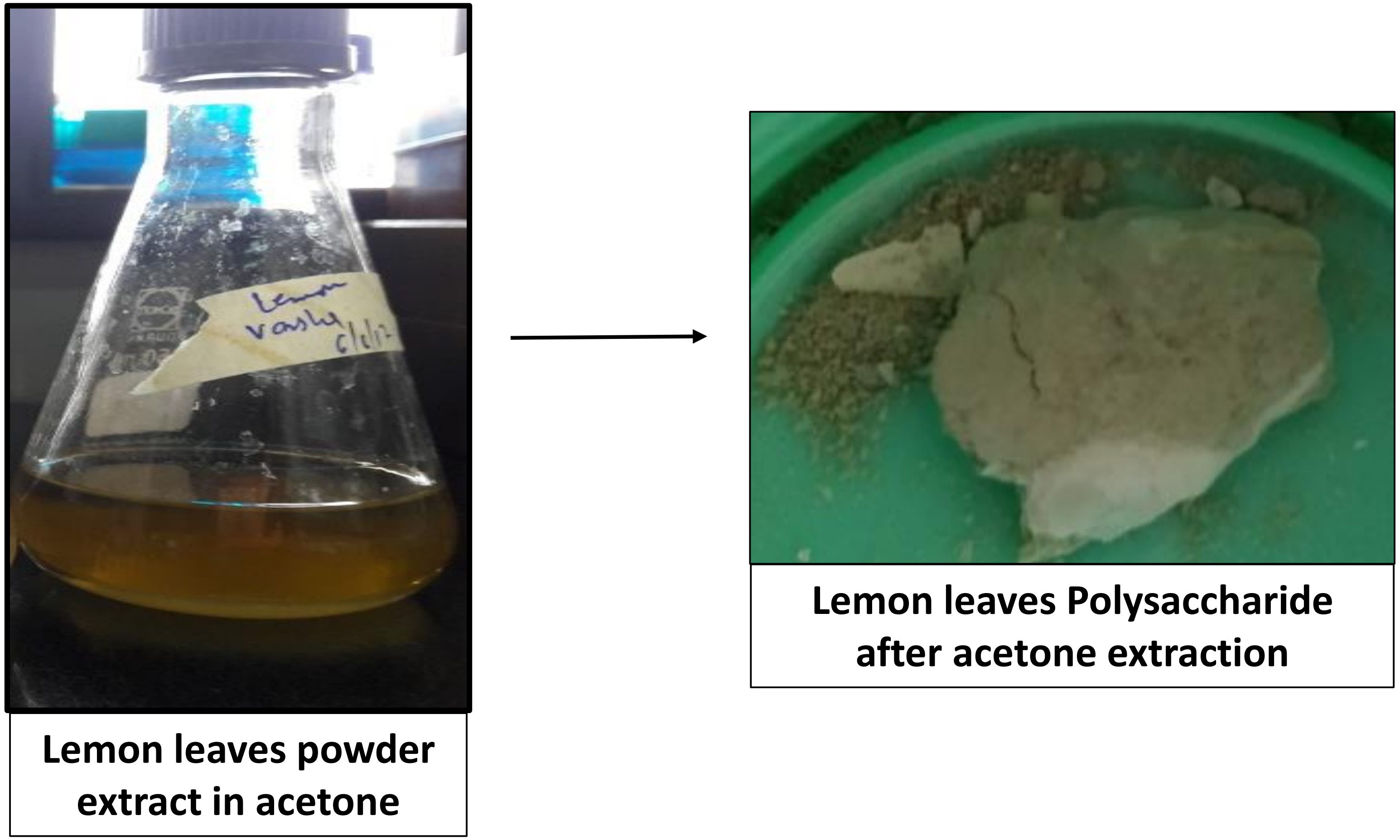

Figure S6. Lemon polysaccharide, extracted from lemon leaves by acetone precipitation.

**Dose of BF-VB2 (%) w/v**	**R^2**	**k**
0.5	0.92	0.0264
1.0	0.93	0.0298
1.5	0.86	0.074
2.0	0.91	0.2578
2.5	0.94	0.0647
3.0	0.90	0.0208

R^2 and k represents regression and slope from the graph

(A)

**Dose of BF-VB2 (%) w/v**	**R^2**	**k**
0.5	0.92	0.0035
1.0	0.70	0.0031
1.5	0.95	0.0077
2.0	0.53	0.0084
2.5	0.98	0.0048
3.0	0.97	0.0029

R^2 and k represents regression and slope from the graph

(B)

Table S1. Pseudo Second order kinetics corresponding k (rate of reaction) and R^2 values for different concentration of BF-VB2 (A). for COD reduction, and (B). for dye decolorization

$$Kaolin\ particles\ left\ after\ flocculation = (A \div B) \times C \qquad (1)$$

Where, A= initial kaolin clay particles (in mg) present in solution

B= the amount (in mL) of kaolin clay solution used for the study

C= F.A. % for this solution

$$Kaolin\ particles\ adsorbed = D \div E \qquad (2)$$

Where, D= value obtained from equitation (1)

E= amount of bioflocculant used (mg) for treating the kaolin clay solution

Equation S1. Calculation to assess the amount of kaolin particles being flocculated by the bacterial bioflocculant BF-VB2.
